# Extraembryonic Origin of Circulating Endothelial Cells

**DOI:** 10.1371/journal.pone.0025889

**Published:** 2011-10-14

**Authors:** Luc Pardanaud, Anne Eichmann

**Affiliations:** 1 Center for Interdisciplinary Research in Biology (CIRB), Collège de France, Paris, France; 2 INSERM U1050, 75005 Paris, France; 3 CNRS UMR 7241, 75005 Paris, France; University of Bristol, United Kingdom

## Abstract

Circulating endothelial cells (CEC) are contained in the bone marrow and peripheral blood of adult humans and participate to the revascularization of ischemic tissues. These cells represent attractive targets for cell or gene therapy aimed at improving ischemic revascularization or inhibition of tumor angiogenesis. The embryonic origin of CEC has not been addressed previously. Here we use quail-chick chimeras to study CEC origin and participation to the developing vasculature. CEC are traced with different markers, in particular the QH1 antibody recognizing only quail endothelial cells. Using yolk-sac chimeras, where quail embryos are grafted onto chick yolk sacs and vice-versa, we show that CEC are generated in the yolk sac. These cells are mobilized during wound healing, demonstrating their participation to angiogenic repair processes. Furthermore, we found that the allantois is also able to give rise to CEC in situ. In contrast to the yolk sac and allantois, the embryo proper does not produce CEC. Our results show that CEC exclusively originate from extra-embryonic territories made with splanchnopleural mesoderm and endoderm, while definitive hematopoietic stem cells and endothelial cells are of intra-embryonic origin.

## Introduction

The existence of circulating endothelial cells (CEC) was demonstrated ten years ago in adult mice [1], [2]. They were shown to reside in the bone marrow and were mobilized during physiological and pathological angiogenesis including tumor growth and heart failure [3], [4]. These findings opened large perspectives and hopes concerning the use of these cells as putative tools to specifically target tumors or damaged tissues. Clinical trials using marrow-derived CEC injected into patients with coronary heart disease have led to a mild improvement in left ventricular ejection fraction and myocardial perfusion [5]–[8], indicating that CEC hold promise for the treatment of disease, but that their contribution to healing of damaged tissue remains to be studied further.

The role of CEC during angiogenesis is debated in the literature [9]: while some studies have shown that CEC are actively mobilized during tumor angiogenesis, integrate the lumen of neovessels [10] and play a role in metastasis dissemination [11], other authors showed that CEC mobilization during tumorigenesis is inefficient [12] and that these cells rarely reach tumor vessels but rather form a niche of bone-marrow-derived hematopoietic progenitors that colonize the vascular wall and contribute to angiogenesis by release of soluble factors [13]–[16]. A recent review on targeted cancer gene therapy using CEC concluded that although feasible, the efficacy of this strategy to control tumor burden has not shown overwhelming success [17].

The true identity of CEC in vivo also still remains uncertain ten years after their discovery. In particular, diverse molecular markers have been used to describe various forms of CEC and progenitors, here collectively referred to as CEC, including bone marrow-derived endothelial progenitor cells (EPC), cord blood-derived EPC, high proliferative potential-endothelial colony forming cells (ECFC), low proliferative potential-ECFC, endothelial outgrowth cells or mesenchymal stem cells [18]. The diversity of adult CEC suggested that they might have multiple potential sites of origin, similar to hematopoietic stem cells (HSC). Alternatively, they might originate from a single source and acquire diversity at later stages.

All known types of CEC share expression of at least some molecular markers with endothelial cells (EC) and hematopoietic cells (HC), reflecting the close developmental relationship between these cell types. In the adult bone marrow and in the embryo, EC, CEC and HSC reside together in stem cell niches. During embryonic development, EC and HC are first formed as ‘hemangioblastic clusters’ of tightly associated precursors in the yolk sac blood islands [19], [20]. Yolk sac hemangioblasts generate a transient wave of extra-embryonic EC and circulating HC of the erythropoietic and macrophage lineages. This transient first wave of HC is later replaced by an intra-embryonic source of definitive HSC [21], defined by their ability to repopulate lethally irradiated hosts after transplantation [22]. Definitive HSC are morphologically conspicuous in the ventral wall of the dorsal aorta, where they bud off the endothelial lining and give rise to HSC [22]. The epithelio-mesenchymal transformation of these ‘hemogenic’ EC is enhanced by shear stress generated by the blood flowing through the aorta and requires NO-production [23]–[25]. Thus, while the yolk sac generates a transient population of EC and HC, definitive HSC are born in close contact to the endothelium of the dorsal aorta, in an intra-embryonic location.

The embryonic site(s) of CEC production had not been determined previously. Using a parabiosis model between chick and quail embryos, we had previously shown that CEC are produced in the embryo prior to bone marrow formation [26]. We had moreover shown that embryonic CEC show hallmarks of adult CEC identified in mouse models, in that they are rare cells sometimes integrated in vessels but mostly located in the interstitium, which participate to neo-angiogenesis processes including wound healing.

We here asked if CEC are first produced in the yolk sac and/or have a double yolk sac as well as intra-embryonic origin. To distinguish between these possibilities, we used a quail-chick chimera model where chick embryos are grafted onto a quail yolk sac and vice-versa. This model was instrumental to demonstrate the intra-embryonic origin of definitive HSC 30 years ago [21]. Quail EC, as well as CEC, can be specifically labeled with the monoclonal antibody QH1 [27]. We found that the yolk sac, but not the embryo proper, gives rise to CEC. Furthermore, constructing half embryo chimeras in which the caudal part of the embryo was of quail origin and the rest of chick origin, we found that the allantois, previously shown to generate CEC when ectopically grafted [28], is also able to give rise to CEC in situ. Thus, using these models, we show here that CEC are generated exclusively in extraembryonic territories made with splanchnopleural mesoderm and endoderm, but not in the embryo proper.

## Materials and Methods

### Yolk sac chimeras

**Figure 1:**
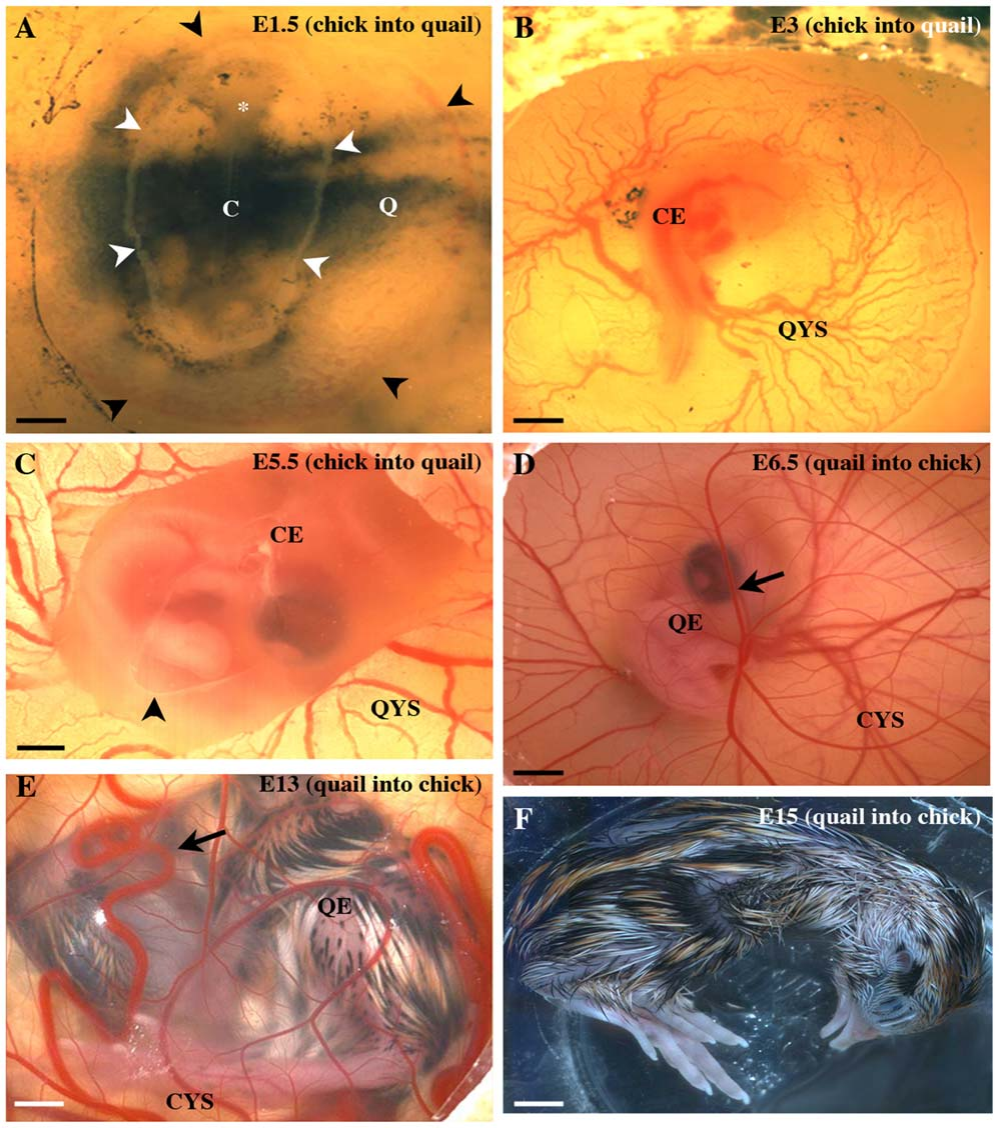
Generation of yolk sac chimeras. A–C) chick embryos on quail yolk sacs; A) A yolk sac chimera just after the operation: in this case, a 10 somite old chick embryo (C) is grafted on a quail yolk sac recipient at the same stage (Q). The black arrowheads show the sinus marginalis, the limit of the quail vascular area. The white arrowheads point to the suture between the chick and quail territories. The asterisk marks the chick head. Bar: 1 mm. B) A yolk sac chimera two days after the operation: in the quail egg a chick embryo (CE) correctly develops and vascular connections with the quail yolk sac (QYS) are normal. Bar: 2 mm. C) A chimera after four days, with a healthy E5.5 chick embryo (CE), wrapped in the amnion (arrowhead) and connected to a quail yolk sac (QYS). Bar: 1.5 mm. D–F) quail embryos on chick yolk sacs; D) After 5 days the quail embryo (QE) developed in connection with the chick yolk sac (CYS). The arrow points to a CAM vessel. Bar: 2 mm. E) An E13 quail embryo (QE) wrapped in its CAM (arrow) develops on a chick yolk sac (CYS). Bar: 7 mm. F) An E15 quail chimera isolated from the chick egg. Bar: 8 mm.

Chick (Gallus gallus, JA57) and quail (Coturnix coturnix japonica) eggs were incubated horizontally for 24h at 38°C until they developed the appropriate somitic stages (s.s.). Yolk sac chimeras [29] were made with a donor and a host at the same s.s. and only embryos that had not yet established circulation (younger than 13s.s.) were used. The donor embryo was isolated together with its yolk sac and cleaned in phosphate buffered saline (PBS)+Ca^2+^Mg^2+^. Using Pascheff scissors the embryo was carefully separated from the yolk sac at the level of the margin separating the two regions. The host was injected with Indian ink (diluted 1/1 in PBS+Ca^2+^Mg^2+^) beneath the blastoderm, and the embryonic territory was removed from the egg. Then the donor embryo was positioned at the surface of the host egg. A crucial point to ensure survival of grafts was to prepare a donor blastodisc just larger than the host counterpart. One drop of PBS+Ca^2+^Mg^2+^ was placed in the hole left after removal of the host embryo to avoid leakage of yolk. The donor embryo was placed at the top of the hole in the proper orientation and the host and the donor tissues were carefully sutured at their margin using two pairs of fine forceps. The string of excess tissues at the surface of the suture was removed with Pascheff scissors, avoiding any leakage around the suture (Fig. 1A). The egg was then sealed with scotch tape and re-incubated for 1 to 13 days (Fig. 1B–F). Both possible combinations, quail embryo on chick yolk sac and chick embryo on quail yolk sac, were generated. Until E6.5, embryos with a part of the yolk sac were fixed overnight at 4°C in Paraformaldehyde 4% (PAF) or Alcohol 100°/Acetic Acid 1% at −20°C, dehydrated, embedded in paraffin, serially sectioned and stained. In older chimeras, pieces of choriallantoic membrane (CAM), meninges, mesentery, aorta, jugular veins and skin were isolated and fixed in PAF. All were processed for in toto immunostaining after overnight PAF fixation as previously described [26]. Bone marrow was retrieved by dissociating long bones. Drops (20ml) of bone marrow cellular suspension in culture medium (DMEM+10% Fetal Calf Serum-FCS-) were placed in Petri Dishes (35mm) and cultured 24h at 37°C, 5% CO_2_. Cultures were fixed in PAF 15mn at room temperature, rinsed in PBS and processed for immunocytochemistry.

### Wound healing assays

On E5 yolk sac chimeras, the chick amnion was sectioned and a wing was exposed at the surface. A deep longitudinal incision was made at the tip of the wing using a microscalpel. The embryos were sacrificed after 3 to 8 hours (n = 20). The two chick wings (wounded and contralateral) were isolated, fixed in Alcohol 100°/Acetic Acid 1% at −20°C, dehydrated, embedded in paraffin, serially sectioned and stained.

### In vitro quail-chick chimeras

As above, pre-circulation quail and chick embryos at the same stage (5–12s.s.) were used. A chick blastodisc, isolated together with its yolk sac was placed in a Petri dish, on a semisolid medium containing 50% agar, 25% yolk, 20% PBS+Ca^2+^Mg^2+^ and 5% penicillin-streptomycin. A quail embryo was carefully isolated from its yolk sac and placed in the Petri dish, close to the chick yolk sac. Using two pairs of fine forceps, a suture was made between the quail and chick territories. The embryos were incubated during 1 to 2 days at 37°C, 5%CO2 then fixed in Alcohol 100°/Acetic Acid 1% at −20°C, dehydrated and embedded in paraffin.

### Half embryo chimeras

The technical approach was the same as above except that we isolated the territory behind the last formed somite from the quail embryo and grafted it into a chick recipient from which the corresponding piece had been removed. The chimeras were incubated until E5.5–E6, a stage where the allantois was well differentiated. Chimeras, allantois and a part of yolk sac were separately fixed in PAF, dehydrated, embedded in paraffin, serially sectioned and stained.

### Staining

Different double/triple stainings were performed using the following markers:

QH1 [27] immunostaining was performed on 7.5 mm paraffin sections or cells as previously described [26]. QH1 (undiluted hybridoma supernatant or 1/1000e ascites -Developmental Studies Hybridoma Bank -DSHB- developed under the auspices of the NICHD and maintained by The University of Iowa, Department of Biology, Iowa City, IA 52242) was visualized using peroxydase- (BioRad) or Alexa 350/488/555- (Invitrogen) conjugated secondary antibodies.

BEN monoclonal antibody (DSHB) stains quail and chick peripheral projecting neurons and hematopoietic precursors [30]. After rehydration, sections were pretreated with 0.025% trypsin at 37°C for 30 minutes. BEN (1/10^e^) was revealed by an Alexa 488/555 goat anti mouse IgG1 (Invitrogen).

LEP100 monoclonal antibody (DSHB) stains avian macrophages [31]. After an incubation in PBS/FCS 3%/Triton 0.1%, LEP100 (1/5) was revealed by an Alexa 488/555 goat anti mouse IgG (Invitrogen).

8F3 monoclonal antibody (DSHB) stains the cytoplasm of all chick cells but does not stain quail cells [32]. 8F3 (1/5) was revealed by an Alexa 488 goat anti mouse IgG1 (Invitrogen).

Polyclonal Rabbit Anti-Human von Willebrand Factor (DakoCytomation) recognizes avian von Willebrand Factor. After rehydration, sections were pretreated with 0.025% trypsin at 37°C for 30 minutes. The antibody was diluted 1/100 in PBS/triton 0.1% and was revealed by an Alexa 555 goat anti rabbit (Invitrogen).

Biotinylated *Sambucus nigra* lectin (1/400 in PBLEC buffer-PBS pH6.8, 1 mM CaCl2, 1 mM MgCl2, 0.1 mM MnCl2, 1% triton) recognizes chick and quail EC (Clinisciences, [33]) but also avian macrophages. The lectin was revealed using Cy3 streptavidin (Amersham).

Biotinylated LEA agglutinin (*Lycopersicon esculentum*, Sigma) labels avian macrophages and venous endothelium [34]. After rehydration, sections were pretreated with 0.025% trypsin at 37°C for 10 minutes. Biotinylated LEA (20 mg/ml in PBS-0.1% triton, overnight at 4°C) was revealed using Cy3 streptavidin (Amersham).

Nuclei were counterstained with glychemalun or Hoechst 33342 after the immunochemistry [26].

### Quantification of QH1^+^ CEC

Observation and counting was done using Leica or Olympus microscopes. For each harvested graft, serial transverse sections were prepared. The volume of embryos or wing buds was calculated using a micrometric scale by measuring the surface of the first section where the tissue was visible plus one section per slide multiplied by the thickness of all sections containing embryonic tissues (n×7.5 mm). The number of quail QH1^+^ EC was counted manually on 1150 sections (x25 objective, final magnification x110, 2300 counted cells). For cell counting in bone marrow cultures, the percentage of 8F3^+^ cells was calculated on 10 different randomly chosen fields per chimera. Statistical analyses were carried out using Mann-Whitney's test.

## Results

### Yolk sac origin of CEC

**Figure 2:**
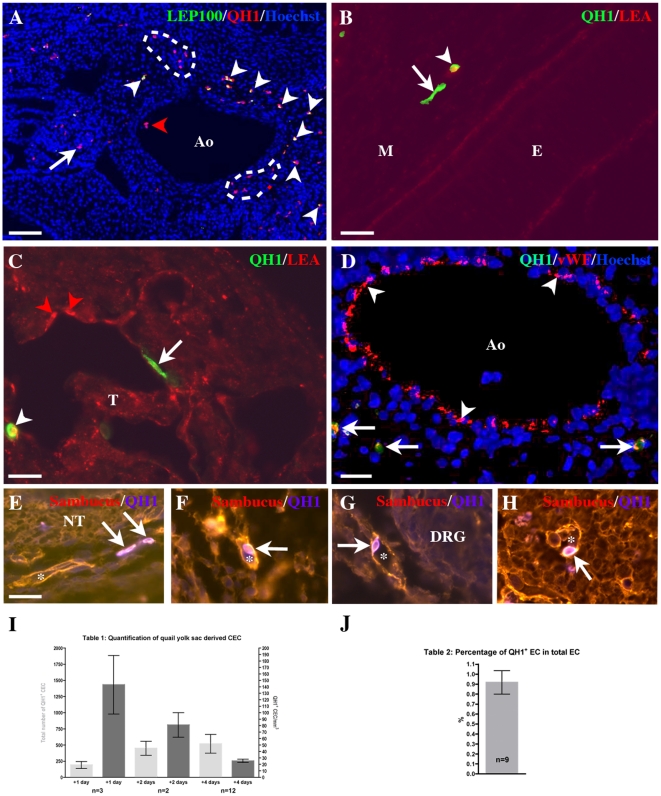
Identification of yolk sac-born QH1^+^ cells in E5.5 chick embryos isolated from yolk sac chimeras. Transverse sections. A) Around the aorta (Ao) a population of QH1^+^ cells was present: the majority is LEP 100^+^ macrophages (arrowheads), but some QH1^+^/LEP100^−^CEC are present (dotted areas, arrow). QH1^+^/LEP 100^−^ CEC are observed in the circulation (red arrowhead). Bar: 40 mm B) Detection of an interstitial QH1^+^/LEA^−^ CEC (arrow) and of a QH1^+^/LEA^+^ macrophage (arrowhead) in the brain mesenchyme (M), close to the neural epithelium (E). Bar: 20 mm. C) A QH1^+^/LEA^−^ CEC (arrow) reaches the endocardium. Note the presence of one quail QH1^+^/LEA^+^ (white arrowhead) and two chick QH1^−^/LEA^+^ (red arrowheads) macrophages. T: trabecula. Bar: 20 mm. D) Identification of three QH1^+^/vWF^+^ CEC in the periaortic mesenchyme (arrows). Note the vWF^+^ endothelium of the aorta (Ao, arrowheads). Bar: 20 mm. E–H) Four examples of vessel-integrated (*) QH1^+^/Sambucus nigra^+^ quail CEC (arrows) in the wing (E), the dermis (F), at the vicinity of the dorsal root ganglia (G, DRG) and in the ventral vascular plexus of the neural tube (H). Bar: 20 mm. I) Table 1: Quantification of quail yolk sac derived CEC in the chick embryo. Counting of QH1^+^ CEC in the embryo shows that the total number of yolk-sac derived CEC (clear gray) increases between 1 and 4 days after grafting. However, as the embryo size expands during the same period, the CEC concentration per mm^3^ of embryonic tissue (dark gray) decreases. J) Table 2: Percentage of CEC that reached host endothelium.

To determine if the yolk sac produces CEC we created yolk sac chimeras in which pre-circulation chick embryos are grafted on yolk sacs from quail embryos at the same s.s. (Fig. 1A, see Methods). As this graft involves replacement of an entire embryo, without damaging the yolk sac of the host, it is technically very challenging, but uniquely suited to study the developmental potential of the yolk sac to generate CEC [29]. Following the onset of blood-flow, cells can circulate from the yolk sac to the embryo, and CEC originating from the quail yolk sac can be traced using the QH1 monoclonal antibody [27], which specifically recognizes quail EC and HC. QH1 also labels non-circulating quail EC in the yolk sac of the chimeras and HC, in particular yolk sac derived macrophages. Quail CEC were distinguished from macrophages using LEA and LEP100 macrophage-specific markers [31], [34]: quail macrophages were QH1^+^/LEA^+^ or QH1^+^/LEP100^+^ while quail CEC were only recognized by QH1 (Fig. 2 A–C). Furthermore quail CEC were identified using specific endothelial markers vWF and Sambucus nigra [26], [34] in addition to QH1.

One hundred and one yolk sac chimeras were constructed (Fig. 1A). Their survival was 38% after one day, 38% after two days and 32% after four days. The oldest embryos we obtained reached 5.5 days of development (stage 28 of Hamburger and Hamilton, HH, [35]). Beyond this stage, all chick embryos (n = 9) died within 12h, probably because the quail yolk sac did not grow large enough to feed the chick embryos, which dramatically increase in size from E5 onwards.

Serial sections of 27 chimeras were prepared, 3 after one day, 2 after two days (Fig. 1B) and 22 after four days (Fig. 1C). In all cases, the chick embryos developed normally in the quail eggs, the morphology and the blood supply was comparable to un-operated embryos and the suture between the chick tissues and the quail yolk sac had become invisible (Fig. 1B, C). The histology of chick embryos also confirmed normal morphogenesis and organogenesis. Numerous yolk sac derived QH1^+^/LEA^+^ macrophages rapidly invaded chick tissues in particular in the head, the limb buds and around the aorta (Fig. 2A). Their morphology was variable, most cells being round (Fig. 2B, C) but in some cases macrophages appeared as thin long cells or star shaped cells (not shown). Most QH1^+^/LEA^+^ macrophages remained isolated in tissues (Fig. 2A–C) but formed aggregates in some cases (not shown). Chick QH1^−^/LEA^+^ macrophages were also identified (Fig. 2C).

Yolk sac-derived CEC colonized the chick tissues as soon as the first day after surgery (Fig. 2). Their number was much less important than the number of invading macrophages (Fig. 2D–H). Quantification showed that the total number of quail CEC increased from day 1 to day 4 after operation (from 192 to 517 cells/embryo, Fig. 2I) but that their density per mm^3^ of embryo decreased with time (from 143 to 26 cells/mm^3^ at day 4 after operation). The density of CEC found at day 4 after operation is equivalent to that previously observed in quail-chick parabioses before the onset of bone marrow formation [26], suggesting that most CEC are generated in the yolk sac (Fig. 2I).

Quail yolk sac CEC were distributed throughout the chick embryos at all stages analyzed; they colonized the meninges and the periphery of the neural tube (Fig. 2B, E), the somatopleural and splanchnopleural mesoderm, the dermis (Fig. 2F), myotome and the dorsal root ganglia (Fig. 2G). During organogenesis, quail CEC were found in the heart (Fig. 2C), the liver, the lung and the limb buds (Fig. 2H). The proportion of interstitial (Fig. 2B, D) and endothelium-integrated (Fig. 2C, E–H) quail CEC was quite similar. Within the endothelium, quail CEC were mostly localized in capillaries and exceptionally in large vessels such as the aorta or the cardinal vein (not shown). The contribution of CEC to the host endothelium was assessed by counting the percentage of QH1^+^ CEC inserted in chick endothelium on 9 E5 yolk sac chimeras (chick embryo on quail yolk sac). The percentage of CEC that participated to host endothelium was ±1% (Fig. 2J), as shown previously for parabioses [36].

To study later stages, in particular the bone marrow, we performed grafting experiments in the reverse combination, quail embryo on chick yolk sac. In all cases, the quail embryos developed normally in the chick eggs, with a morphology and a blood supply similar to un-operated embryos and the suture between the chick tissues and the quail yolk sac was invisible (Fig. 1D). In this combination, the survival of quail embryos was possible beyond five days after the operation, most likely because the larger size of the chick yolk sac allowed sufficient blood and nutrient supply to sustain growth of the quail embryo (Fig. 1E, F).

The resulting chimeras (5/33) were autopsied when they reached E13 (1/5, Fig. 1E)–E15 (3/5, Fig. 1F). One chimera was left up to E15 to hatch. The embryo was perfectly developed but did not manage to hatch, probably because the chick shell was too thick to break for the quail embryo (not shown).

**Figure 3:**
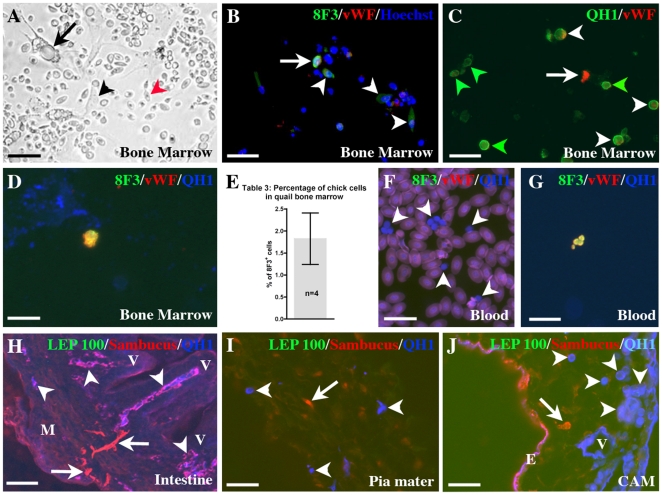
Distribution of chick yolk sac-born CEC in E15 quail chimeras. A–D and F) bone marrow cultures. A) Aspect of a quail bone marrow culture after one day; Adipocytes with oil vacuoles (arrow), fibroblasts with filopodia (black arrowhead) and nucleated erythrocytes (red arrowhead) are easily identified. The round cells present in the culture are HC. Bar: 40 mm. B) 8F3/vWF double staining identifies chick CEC (arrow) among 8F3^+^/vWF^−^ chick cells (arrowheads). Bar: 20 mm. C) The QH1/vWF double staining shows a chick CEC (arrow) among quail EC (white arrowheads) and HC (green arrowheads). Bar: 20 mm. D) A QH1^−^/vWF^+^/8F3^+^ CEC is detected in a bone marrow smear. Bar: 10 mm. E) Table 3: Percentage of chick cells invading the bone marrow in the oldest yolk sac chimeras. The number of 8F3/Hoechst cells is compared with the total cell population in cultures. F**)** Presence of QH1^+^ HSC among nucleated erythrocytes in a blood smear. Bar: 20 mm. G) Identification of a group of QH1^−^/vWF^+^/8F3^+^ CEC in a blood smear. Bar: 20 mm. H) Triple staining on a transverse section through the intestine illustrating the participation of chick QH1^−^/Sambucus^+^/LEP100^−^ CEC (arrows) to the vascular plexus of smooth muscle layers (M), beneath a villus (V). The quail host vessels are QH1^+^/Sambucus^+^/LEP100^−^ and appear purple (arrowheads). Bar: 40 mm. I) On a pia mater section, an interstitial QH1^−^/Sambucus^+^/LEP100^−^ CEC (arrow) is identified among QH1^+^ HC (arrowheads). Bar: 20 mm. J) An interstitial QH1^−^/Sambucus^+^/LEP100^−^ CEC (arrow) on a CAM section close to a QH1^+^ vessel (V) and to QH1^+^ HC (arrowheads). Note that the endodermal layer (E) of the CAM is underlined by Sambucus and QH1 and appears purple. Bar: 20 mm.

On 4 of these chimeras, quail bone marrow was collected from long bones and cultured overnight before fixation. In culture, fibroblasts, red blood cells and adipocytes were easily identified with bright field observations (Fig. 3A). If CEC were produced in the yolk sac, these chimeras should contain chick CEC in the quail bone marrow. To identify chick cells, we used the 8F3 monoclonal antibody specific for the cytoplasm of all chick cells (Fig. 3B, D, F, S1A, B). The percentage of chick cells in the bone marrow, calculated using 8F3/Hoechst double staining, was around 2% (Fig. 3E). Bone marrow cultures and bone marrow smears indeed contained 8F3^+^/vWF^+^ (Fig. 3B) and QH1^−^/vWF^+^ (Fig. 3C, D) cells, attesting the presence of chick CEC in the quail bone marrow. As expected, other chick cells were present as well.

To characterize 8F3^+^ cells, different double stainings were performed. 8F3/LEA double staining showed that some chick cells were not macrophages (Fig. S1B). QH1/BEN double staining permitted to show that the major part of QH1^+^ cells were hematopoietic progenitors (Fig. S1C). QH1^+^/vWF^+^ as well as QH1^+^/BEN^−^ cells (Fig. 3C, S1C) were probably quail EC coming from sinusoids present in the bone marrow. Nevertheless, we cannot exclude they were quail CEC coming from an unknown embryonic territory.

Finally, the analysis of blood smears taken from host quails permitted to find QH1^+^ HSC (Fig. 3F) and to identify a few QH1^−^/8F3^+^/vWF^+^ chick CEC (Fig. 3G).

We also histologically checked the presence of chick CEC in organs. As the 8F3 antibody did not work on paraffin section in our conditions, we analyzed sections with QH1/LEP100/Sambucus nigra staining which permitted to recognize QH1^−^/LEP 100^−^/Sambucus^+^ chick CEC. The CEC were found either integrated in vessels (Fig 3H) or interstitially located (Fig. 3I, J). The various observations made on later developmental stages confirmed the ability of the yolk sac to produce CEC.

The organs were also invaded by chick macrophages. In the meninges, in toto QH1/8F3 immunostaining showed a lot of 8F3^+^ chick cells located close to QH1^+^ capillaries of the pia mater (Fig. S2A). At higher magnifications, 8F3^+^ filopodia projecting towards QH1^+^ capillaries were sometimes observed (Fig. S2B). On section, a QH1/Sambucus/LEP100 staining confirmed the macrophage identity of these cells (QH1^−^/Sambucus^+^/LEP100^+^, Fig. S2C). In the skin, chick cells were identified as macrophages (Fig. S2D, E). Chick macrophages were also observed in internal organs as the heart (Fig. S2F). In toto immunostainings showed that no 8F3^+^ chick cells were present in large vessels (aorta and jugular vein) as well as in aortic vasa vasorum (Fig. S2G–I).

### Yolk sac-derived CEC are mobilized during wound healing

**Figure 4:**
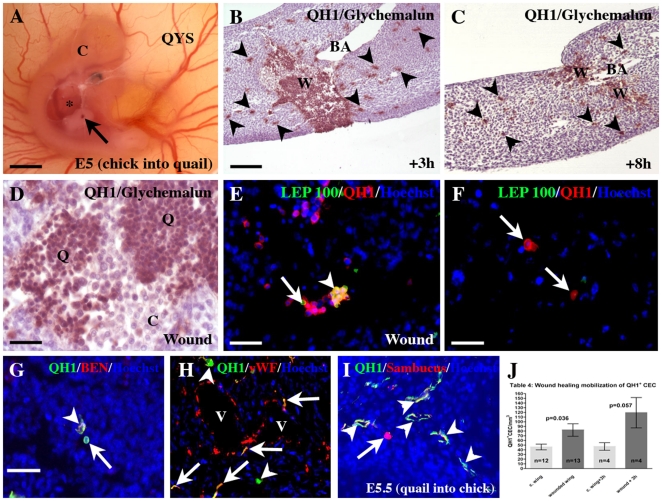
CEC mobilization during wound healing. A) An E5.5 chick embryo (C) developed on a quail yolk sac (QYS). A wound is made in the right wing bud (arrow), which is accessed through a hole in the amnion (*). Bar: 1.2 mm. B, C) Longitudinal sections of wounded wings. Three hours after the operation (B), at the level of the brachial artery (BA), the wound (W) is invaded by an important contingent of chick (blue) and QH1^+^ quail cells (brown). After 8h (C), the rapid healing of the wound (W) is obvious. Note that numerous QH1^+^ cells (arrowheads) are present in the wing mesenchyme (B, C). Bar: 85 mm. D) High magnification of the mixed population of brown quail cells (Q) and blue chick cells (C). Bar: 20 mm. E) QH1/LEP 100 double staining permits to discriminate between QH1^+^/LEP 100^+^ macrophages (arrowhead) and QH1^+^/LEP 100^−^ cells (arrow) in the wound. Bar: 40 mm. F–H) Identification of QH1^+^ cells in the mesenchyme of wounded wings. F) QH1^+^ cells are not macrophages (arrows). Bar: 20 mm. G) A QH1^+^/BEN^+^ HC (arrowhead) is identified close to a non hematopoietic QH1^+^/BEN^−^ cell (arrow). H) In the vicinity of a QH1^−^/vWF^+^ chick vessel (V), QH1^+^/vWF^+^ EC (arrows) are present with QH1^+^/vWF^−^ HC (arrowhead). I) In the reverse combination, quail embryo grafted on chick yolk sac, a section of QH1^−^/Sambucus^+^ chick vessel (arrow) present among the QH1^+^/Sambucus^+^ quail vascular plexus (arrowheads). Bar: 40 mm in F, 30 mm in G–I. J) Table 4: Mobilization of yolk sac derived CEC during wound healing. A wound on wings induces a statistically significant mobilization of CEC (dark gray) by comparison with CEC concentration in contralateral limbs (clear gray). This mobilization is effective as soon as 3h after wounding (right part of the table).

We next tested if yolk sac derived CEC could participate to repair processes such as wound healing. An incision was made in a wing of E5.5 chimeras (Fig. 4A) and the embryos (n = 17) were sacrificed 3–8 hours later. Nineteen chimeras concerned chick embryos grafted on quail yolk sacs, one experiment was performed in the reverse combination. Histological observations showed that the healing process took place rapidly as the wound depth was dramatically reduced between 3 and 8 hours (Fig. 4B, C). An important contingent of circulating cells invaded the wound cavity as soon as 3hours after the operation (Fig. 4B, D). These cells were a mix of QH1^−^ chick cells and QH1^+^ quail cells (Fig. 4D). The major part of quail cells was QH1^+^/LEP100^+^ macrophages (Fig. 4E) but QH1^+^/LEP100^−^ cells were observed (Fig. 4E): as the yolk sac is known to produce erythrocytes and macrophages, QH1^+^/LEP100^−^ cells present in the wound cavity could be CEC since QH1 does not recognize erythrocytes. The invading QH1^+^ cells were distributed throughout the wounded wing, even in areas distant from the wound site (Fig. 4B, C). In the wing mesenchyme, part of the invading quail cells were not macrophages (Fig. 4F) and did not belong to the hematopoietic lineage (Fig. 4G). Furthermore, QH1/vWF and QH1/Sambucus nigra double stainings permitted to clearly identify CEC among the invading population (Fig. 4H, I). These CEC were interstitially located or integrated in capillary endothelia (Fig. 4H, I) in equivalent proportion. Counting of QH1^+^ CEC showed a statistically significant higher concentration in wounded wings compared with the number of QH1^+^ CEC in contralateral wings (Fig. 4J). Interestingly, this increase of QH1^+^ CEC in wounded limbs was effective as soon as 3h after the surgery (Fig. 4J), suggesting that the mobilization of QH1^+^ CEC during wound healing was a rapid process.

### The embryo proper does not produce CEC

To determine if the embryo proper was able to produce CEC, we constructed yolk sac chimeras in which a quail embryo was grafted on a chick yolk sac in a chick egg. In these conditions, the capacity of the embryo to produce CEC could be attested by the presence or the absence of QH1^+^ EC in the chick yolk sac.

Twenty-seven yolk sac chimeras were constructed. Their survival was 44% after one day, 44% after two days, 33% after four days and 50% after five days. The oldest quail embryos we sacrificed reached 6.5–7 days of development (stage 23 Zacchei, 1961 [37]; Fig. 1D).

Serial sections of 11 chimeras were prepared, 3 after one day, 3 after two days, 3 after four days and 2 after five days (Fig. 1D).

**Figure 5:**
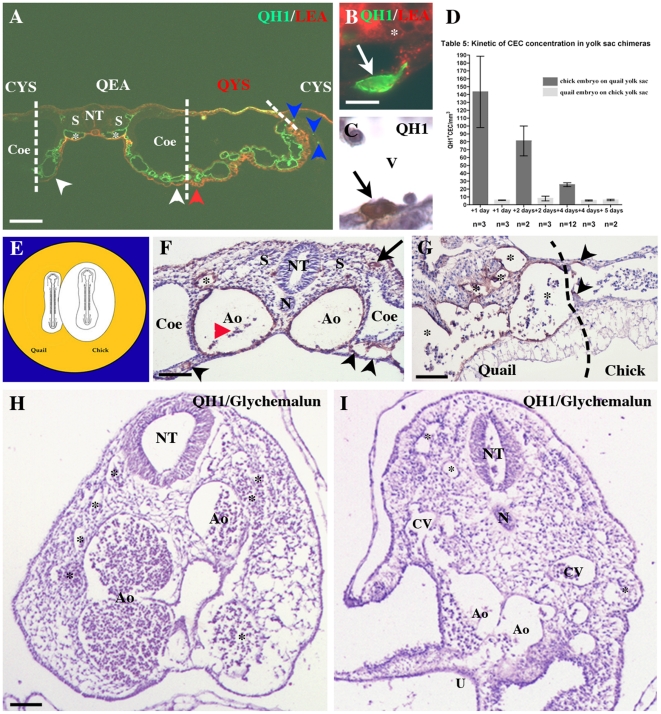
Absence of embryo-derived CEC. A) Transverse section through the trunk of a yolk sac chimera (quail embryo/chick yolk sac) one day after the operation. On the right, the grafted quail territory in which all the vessels are QH1^+^ (green) includes the embryonic area (QEA) and a part of the quail yolk sac (QYS). The distinction between the two regions is histologically possible comparing their respective unspecific LEA-stained endoderm: the endoderm of the embryonic area (white arrowheads) is thinner than the yolk sac endoderm (red arrowhead). On both sides, the quail tissues are well associated with the chick yolk sac (CYS). The blue arrowheads point to three isolated QH1^+^ cells migrating in chick yolk sac vessels. Coe: coelom; NT: neural tube; S: somite; *: aorta. Bar: 110 mm. B) A QH1^+^/LEA^−^ CEC (arrow) interstitially migrates at the surface of the chick yolk sac endoderm (*). C) A QH1^+^ CEC (arrow) reaches the endothelium of a yolk sac vessel (V). Bar: 20 mm in B and C. D) Table 5: Comparison of CEC number in quail yolk sac-chick embryo chimeras (dark gray) and chick yolk sac quail embryo chimeras (clear gray). This histogram shows that in the combination chick yolk sac/quail embryo this number is constant and residual (clear gray) by comparison with CEC concentration in the opposite combination (dark gray). E) Scheme of the in vitro quail-chick chimera procedure: on a semi-solid medium (orange) a quail embryonic territory, completely isolated from its yolk sac, and a whole chick blastoderm (embryonic + yolk sac areas) are placed side by side to permit the establishment of vascular connections. F–I) QH1 immunohistochemistry on transverse sections of in vitro chick-quail chimeras 1.5 day after the operation: F) The quail embryo is viable as shown by 1) chick blood cells (red arrowhead) in the aortae (Ao), which attested that a circulation is established with the chick territory, and 2) the presence of QH1^+^ vessels in the somatopleural (arrow) and splanchnopleural mesoderms (arrowheads). Coe: coelom; NT: neural tube; N: notochord; S: somite; *: cardinal vein. Bar: 50 mm. G) Region of contact between the two embryos: the broken line delimits the quail territory on the left, with QH1^+^ endothelia (*), and the chick territory on the right, with QH1^−^ endothelium: note the presence of two QH1^+^ EC which interstitially migrate in the proximal part of the chick vessel (arrowheads). Bar: 50 mm. H, I) Absence of QH1^+^ cells illustrated on two different chick embryos: H) in the hindbrain; I) in the umbilical region. Ao: aorta; CV: cardinal vein; N: notochord; NT: neural tube; U: umbilic; *: vessels. Bar: 90 mm in H, I.

Histological observations of chimeras, one day after the graft, showed that a small rim of quail yolk sac was always grafted together with the quail embryo, at least on one side (Fig. 5A). The contaminating quail yolk sac territory was easily identified on sections by the aspect of the unspecific LEA-staining of the endoderm, which appeared thicker than the endoderm of the embryonic region (Fig. 5A) as previously described [38]. If this contamination had no importance when the yolk sac potentiality was studied, it had to be taken into account when the embryo capacity was studied. Nevertheless, due to the little contaminating yolk sac we observed, we decided to analyze the chimeras.

The vascularization of grafted quail embryos appeared normal as attested by continuous QH1 immunolabeling of the aorta, the splanchnopleural and somatopleural vessels (Fig. 5A) and the endocardium (not shown). Transverse sections of chick yolk sacs showed the presence of round QH1^+^ HC in the lumen of the vitelline vessels (Fig. 5A). A few QH1^+^/LEA^−^ CEC were interstitially located (Fig. 5B) or integrated in endothelia (Fig. 5C). Calculating the concentration of QH1^+^/LEA^−^ CEC in chick yolk sacs, we found a very low number by comparison with number of CEC found in the reverse combination (Fig. 5D) and this concentration did not vary during all stages examined (Fig. 5D). In these conditions, we could not exclude that the QH1^+^/LEA^−^ CEC observed in chick yolk sacs came from the piece of contaminating quail yolk sac grafted with the quail embryo. Indeed, the less contaminated yolk sac we grafted, the less QH1^+^ CEC we observed in chick yolk sacs, suggesting that these cells came from the contaminating quail yolk sac and not from the quail embryo proper.

Attempts to perform the same experimental protocol by grafting quail embryos totally devoid of yolk sac tissue were never successful, as the suture between quail and chick territories was impossible to maintain due to the high tension between tissues at this level (not shown).

To palliate this technical difficulty, we created in vitro chimeras consisting of a chick embryo with its yolk sac (10–13 s.s.) placed next to a quail embryo (9–12 s.s.) without its yolk sac, on a semisolid medium (Fig. 5E). In these conditions, the suture between the edges of tissues was correctly maintained, the tension forces being less important on the semisolid medium. We previously showed that in vitro chimeras could survive, could be studied during two days and that quail CEC could colonize chick territories [26]. On 10 associations, 4 were analyzed: the quail and chick embryos respectively reached 15–20 s.s. and 15–23 s.s.. As quail embryos were unable to produce primitive HC due to the absence of their yolk sac, their survival attested the presence of vascular anastomoses with the chick territory allowing to chick HC to feed the quail embryo: the histology confirmed the presence of chick primitive HC in the lumen of the quail vessels (Fig. 5F, G). Morphologically, quail embryos developed a normal vascular tree (Fig. 5F) with functional heart and aorta. At the level of the quail-chick junction, vascular connections were identified between quail and chick vessels (Fig. 5G) and some QH1^+^ EC could interstitially migrate in the endothelium of proximal chick yolk sac vessels (Fig. 5G). Analysis of the distribution of QH1^+^ cells in chick embryos showed absence of quail CEC in all tissues and endothelia (Fig. 5H, I). Thus, using in vitro chimeras to study the ability of the embryonic territory to generate CEC, we show that the embryo *per se* is unable to produce these cells.

### Is the yolk sac the only appendage producing CEC?

It was previously shown that the allantois was able to produce CEC colonizing the bone marrow when ectopically grafted in the coelom [28], but it remained to be determined if it had it the same capacities in vivo. To resolve this point, we created, in chick eggs, half embryo chimeras before the onset of circulation (13s.s.). This surgical technique permitted to discover that HSC destined to colonize intraembryonic organs arise in the whole embryo exept the prospective head-neck region [39].

**Figure 6:**
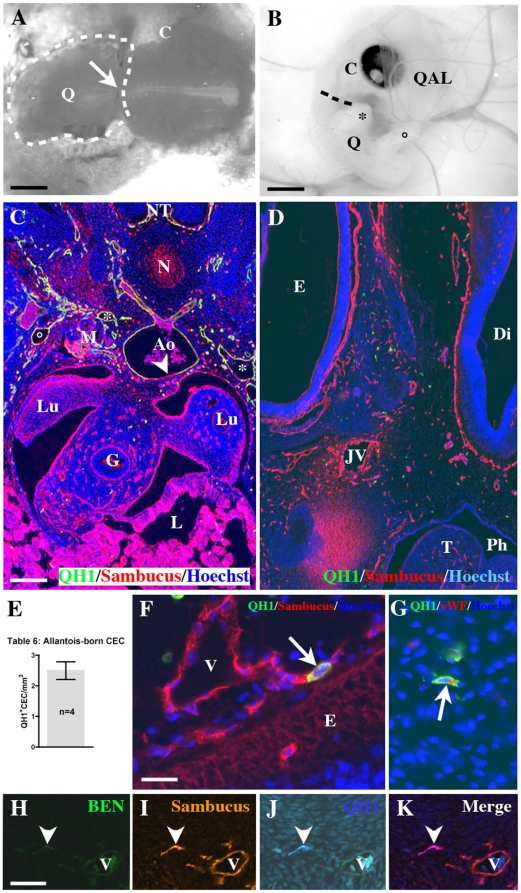
half embryo chimeras. A) A 9 s.s. half embryo chimera just after the operation: the dotted region corresponds to the grafted quail territory (Q) caudally to the last formed somite. The rest of the chimera is chick (C). Note that the quail and chick neural tubes are aligned (arrow). Bar: 1 mm. B) A half embryo chimera 4 days after the operation: the embryo is well developed with its wing (*) and limb (°) buds; the dotted line shows the limit between chick (C) and quail (Q) territories. The quail allantois (QAL) is present above the head. Bar: 2 mm. C) A cross section at the truncal level showing the chick intrinsic QH1^−^/Sambucus^+^ vascularization in the gut (G) and the lungs (Lu). Dorsally, the vascular tree is quail, QH1^+^/Sambucus^+^, in particular around the neural tube (NT), the cardinal veins (*) and the umbilical vein (°). Note that the aortic endothelium (Ao) is chimeric with a part of chick EC located ventrally (arrowhead). Sambucus also labels basement membrane of epithelia in gut, lung and liver (L). N: notochord; M: mesonephros. D) A cross section in the diencephalon. All the vascular network is chick, i. e., QH1^−^/Sambucus^+^ in particular around the right eye (E), the diencephalic vesicle (Di) and the right jugular vein (JV). The green dots are QH1^+^ HC. Ph: Pharynx; T: tongue. Bar: 80 mm in C, D. E) Table 6: Counting of quail CEC identified in chick territories of half embryo chimeras. Less than 3 QH1^+^ CEC/mm^3^ are present. F) Close to the diencephalic epithelium (E), a QH1^+^/Sambucus^+^ CEC (arrow) is integrated in a chick QH1^−^/Sambucus^+^ vessel (V). G) Identification of a QH1^+^/vWF^+^ CEC (arrow) interstitially located in the diencephalic mesenchyme. Bar: 20 mm in F, G. H–K) QH1/Sambucus/BEN triple staining confirms the endothelial nature of a quail purple CEC (arrowhead) in the diencephalic mesenchyme. Bar: 40 mm in H–K.

In these chimeras, the posterior part of a quail embryo, behind the last formed somites, replaced its chick counterpart (Fig. 6A). This surgery produced chimeras with a quail allantois developing in the hind gut region. The resulting chimeras (5/37, Fig. 6B) were autopsied 4 days after the operation at E5.5–6 (stages 27–28HH), 2.5 days after the establishment of vascular connections between the embryo and the allantois (St19HH, [28]). The chimeras developed correctly and the dorsal limit between chick and quail territories was identified by a dilatation at the level where neural tubes fused, sometimes involving the presence two tubes side by side (Fig. S3A). As the grafts were performed before blood circulation, the tissues developed from the quail territory included the allantois (Fig. S3B), the wing (Fig. S3C), limb and tail buds, the body wall and the dorsal structures such as the neural tube (Fig. 6C). In these territories the vascular plexus was QH1^+^ (Fig. 6C, S3A–C). Interestingly, the viscera came from the chick (Fig. 6C). These observations confirmed pioneer fate map experiments showing that, until the level of the 15^th^ somite, the lateral endomesoderm participates to the formation of the digestive tract, but not to the hind gut [40]. Furthermore, as shown previously, the vascular plexus of viscera was chick and QH1^−^ ([41], [42]; Fig. 6C). Heart and lungs (Fig. 6C) had also a chick origin. Rostrally to the wing level, the tissues in the head were chick (Fig. 6D). The vascular plexus became QH1^−^/Sambucus nigra ^+^ (Fig. 6D) and in the territories which were at the boundary between chick and quail, chick EC progressively replaced the QH1^+^ endothelial plexus as in the aortic endothelium (Fig. 6C). Finally, the yolk sac was chick and vascularized by QH1^−^ EC (Fig. S3D).

Concerning CEC, as we found that the embryonic territory was unable to produce these cells (see above), the identification of QH1^+^CEC in chick tissues would mean that these cells came from the allantois. A counting of CEC showed a concentration of 2–3 QH1^+^ CEC/mm^3^ (Fig. 6E). In the chick head we could detect QH1^+^/vWF^+^, QH1^+^/Sambucus nigra^+^ or QH1^+^/Sambucus^+^/BEN^−^ CEC, integrated in endothelia (Fig. 6F) or interstitially located (Fig. 6G–K).

In these chimeras as the limit between chick and quail territories was located rostrally to the wing level, all definitive HSC that arose in the truncal ventral aortic region were quail, thus QH1^+^ (Fig. 6C, D) and were found in chick territories (Fig. S3E, F).

In conclusion, using half embryo chimeras, we showed that the allantois produced CEC in situ and may generate QH1^+^ EC found in the bone marrow of older yolk sac chimeras in addition to the yolk sac.

## Discussion

In this study, we use yolk sac and half embryo chimeras to demonstrate that CEC are generated in extraembryonic appendages, the yolk sac and the allantois, and travel through the circulation to reach embryonic vessels in most organs as well as the bone marrow. The density of CEC in these chimeras was measured by counting the number of quail yolk sac derived QH1^+^ CEC per mm^3^ of embryonic tissue (Fig. 2I, 6E). We have previously demonstrated the existence of embryonic CEC using quail-chick parabiosis, where two embryos are grown inside one eggshell, allowing fusion of their choriallantois membranes and blood circulation from one embryo to another [26]. CEC number in yolk sac and half embryo chimeras is equivalent to that previously observed in quail-chick parabioses prior to bone marrow formation, indicating that all CEC are generated in the yolk sac and the allantois. We previously showed that in older embryos only 5% of CEC were found in endothelia [26]; active angiogenesis early in development apparently requires larger numbers of CEC integrating into vessels.

Using yolk sac chimeras, Dieterlen-Lièvre and Martin [21] discovered that definitive HSC are not generated in the yolk sac, but in the embryo proper. These findings have since been confirmed in mice and humans [22], [43]. The yolk sac produces a transient wave of HC, mostly erythrocytes and macrophages, which are later replaced by a new population of HSC born in the para-aortic splanchnopleura and the ventral wall of the aorta [21]. Some of the yolk sac macrophages invade the embryonic central nervous system and differentiate to microglia [44], [45]. Histological observations have suggested that all microglial cells derive from yolk sac macrophages [44], [46]. Recently, this has been confirmed using lineage-tracing in mice [45]. Taken together with the findings reported here, these observations suggest that the yolk sac, a transient extra-embryonic appendage, gives rise to two cell types persisting in adult, which are microglial cells and CEC.

Constructions of yolk sac chimeras where a quail embryo was grafted onto a chick yolk sac showed that the embryo proper is unable to give rise to CEC. These experiments were particularly challenging technically, as the quail embryo was hard to isolate completely from yolk sac territory and in vitro chimeras had to be generated. However, in spite of the technical difficulties, definitive quail HSC and endothelium were readily seen in these chimeras. Moreover, quail-derived CEC were only found in the chick yolk sac when a piece of contaminating yolk sac had been grafted, and totally absent in the in vitro associations. As quail-chick parabiosis experiments had shown that the density of CEC in the yolk sac and intra-embryonic tissue was similar [26], these results lead us to conclude that the embryo proper cannot give rise to CEC.

Previous studies using quail-chick chimeras had suggested that the allantois, another extra-embryonic appendage, could produce CEC able to colonize the bone marrow after heterotopic grafting into the coelom [28]. Both the yolk sac and the allantois are purely splanchnopleural appendages, i.e., formed from endoderm and splanchnopleural mesoderm, and have hemangiopoietic capacity, i.e., they can give rise to EC and HC. To test whether the allantois preserves its hemangiopoietic capacity in situ, we constructed half embryo chimeras and demonstrated that the allantois is able to give rise to CEC. As intraembryonic splanchnopleural mesoderm can produce EC and HSC, but not CEC, these data suggest that instructive cues from extraembryonic endoderm might trigger CEC formation as previously shown during hematopoietic development [47], [48].

In mice, the allantois is very rudimentary and the placenta, which has hemangiopoietic capacity, replaces its function [49]. Whether CEC are generated in extraembryonic territory, including yolk sac and placenta in mice remains to be experimentally addressed. As placental tissue is accessible, it would be an attractive source of CEC in addition to HSC [50].

Our results indicate that CEC are generated in the yolk sac and the allantois, suggesting that they are originating from few sites of production and that they acquire diversity during later embryonic stages, perhaps after homing to the bone marrow. The bone marrow provides a suitable environment for the multiplication of CEC, as CEC density in embryonic tissues increases significantly after bone marrow arises in quail-chick parabiosis [26]. Factor(s) inducing CEC multiplication in the marrow and inhibiting CEC multiplication in tissues remain to be identified. Preliminary data suggest that VEGF could play a role in the migration of yolk sac-derived CEC (LP and AE, unpublished data), but additional work is required to identify mechanisms controlling embryonic CEC behavior.

Although they are rare in embryonic tissues, CEC can be mobilized during acute angiogenesis process, i.e., wound healing. Previous experiments using parabiosis had demonstrated that CEC numbers increase significantly after induction of neovascularization by wounding or by grafting of an organ on the CAM, which is then vascularized [26]. CEC also participated in small numbers to vascularization of tumors grafted onto the chick CAM of parabioses [36]. In all cases, they were found in part outside of vessels, suggesting that rather than contributing directly to neovessel formation, they stimulate angiogenesis by release of soluble factors [13]–[15]. This simple model thus reproduces results obtained in mice and reveals an indirect contribution of CEC to neovascularization.

## Supporting Information

Figure S1 **Identification of chick HC in the bone marrow.** A) Two 8F3^+^ chick cells (arrow) observed with QH1^+^ cells among a majority of QH1^−^/8F3^−^ quail population. Bar: 20 mm. B) Two 8F3^+^/LEA^−^ chick cells (arrows) and two 8F3^−^/LEA^+^ quail macrophages (arrowheads) are visible in this field. C) QH/BEN staining identifies double stained HC and one QH1^+^/BEN^−^ cell (arrowhead). Bar: 20 mm. Bar: 20 mm in A–C.
(TIF)

Figure S2 **Distribution of chick yolk sac-born macrophages in E15 quail chimeras.** A) In toto double immunostaining showing 8F3^+^ cells (green) invading the QH1^+^ vascular plexus of the pia mater. Bar: 20 mm. B) High magnification of a 8F3^+^ cell showing a thin filopodial extension (arrow) towards the vascular plexus. Bar: 7 mm. C) Triple staining on a section through the pia mater identifying a chick QH1^−^/Sambucus^+^/LEP100^+^ macrophage (arrow) among QH1^+^ HC (arrowheads). Bar: 20 mm. D) In toto double immunostaining in the skin showing a chick cell (arrowhead) present among QH1^+^ cells. Bar: 20 mm. E) Skin section with a triple staining identifying a chick QH1^−^/Sambucus^+^/LEP100^+^ macrophage (arrow) among QH1^+^ HC (arrowheads). Bar: 20 mm. F) Transverse section through the heart with two chick QH1^−^/Sambucus^+^/LEP100^+^ macrophages (arrows) close to a QH1^+^ coronary vessel (V). Bar: 20 mm. G–I) In toto QH1/8F3 double staining in large vessels does not identify 8F3^+^ chick cells in the quail aortic endothelium (G), the quail aortic vasa vasorum (H) and the quail jugular vein endothelium (I). Note the presence of QH1^+^ HC at the aortic luminal surface (G, orange dots). Bar: 30 mm in G and I, 10 mm in H.
(TIF)

Figure S3 **Half embryo chimeras.** A) This cross section illustrates the junction region between quail and chick territories where quail (QNT) and chick (CNT) neural tubes overlap. In the quail region QH1^+^ vessels are present. In the chick territory, a part of the neural tube is vascularized by quail EC that have migrated interstitially. The QH1^+^ dots on the left are quail HC. B) Section of the quail allantois vascularized by QH1^+^ vessels. C) QH1^+^ vessels present in the quail wing bud. D) Section of the chick yolk sac with QH1^−^ vessels in which QH1^+^ HC (arrows) are observed. E) Presence of a quail QH1^+^/BEN^+^ HC (arrow) in the rhombencephalic mesenchyme together with a chick QH1^−^/BEN^+^ HC (arrowhead), probably a macrophage. Note that BEN stains the neuronal plexus (*) in the epithelium (E). F) LEP100^+^ macrophages detection in the diencephalic mesenchyme. One double stained QH1^+^/ LEP 100^+^ quail macrophage (arrow) is seen among chick ones (arrowheads). Bar: 40 mm in A–F.
(TIF)
