# Supplementary figures and images for: Extraembryonic Origin of Circulating Endothelial Cells
Source: PLoS One. 2011 Oct 14;6(10):e25889. doi: 10.1371/journal.pone.0025889 (PMC3195083; doi:10.1371/journal.pone.0025889)

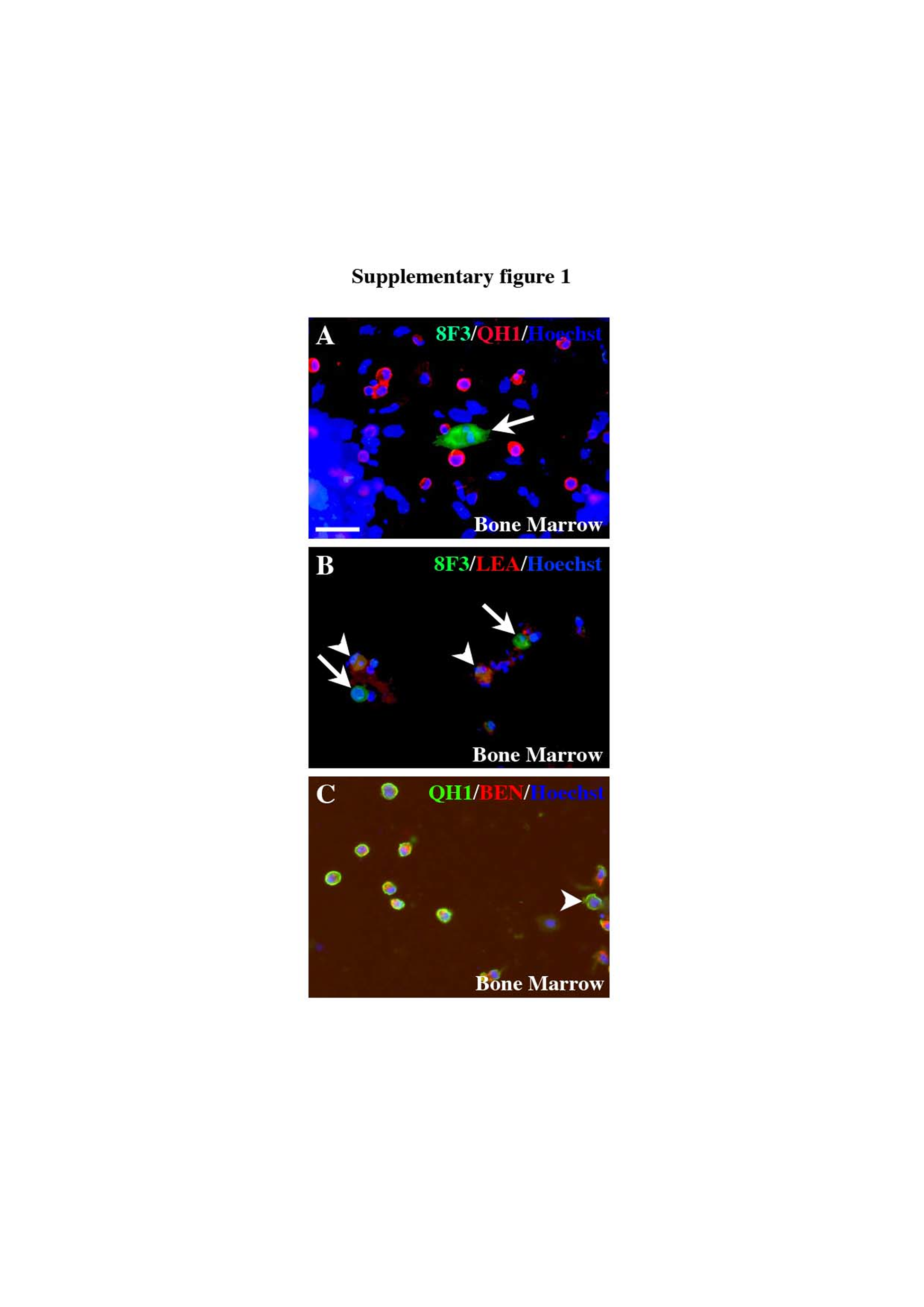

Supplement: Figure S1 — Identification of chick HC in the bone marrow. A) Two 8F3+ chick cells (arrow) observed with QH1+ cells among a majority of QH1−/8F3− quail population. Bar: 20 mm. B) Two 8F3+/LEA− chick cells (arrows) and two 8F3−/LEA+ quail macrophages (arrowheads) are visible in this field. C) QH/BEN staining identifies double stained HC and one QH1+/BEN− cell (arrowhead). Bar: 20 mm. Bar: 20 mm in A–C. (TIF) [file pone.0025889.s001.tif]

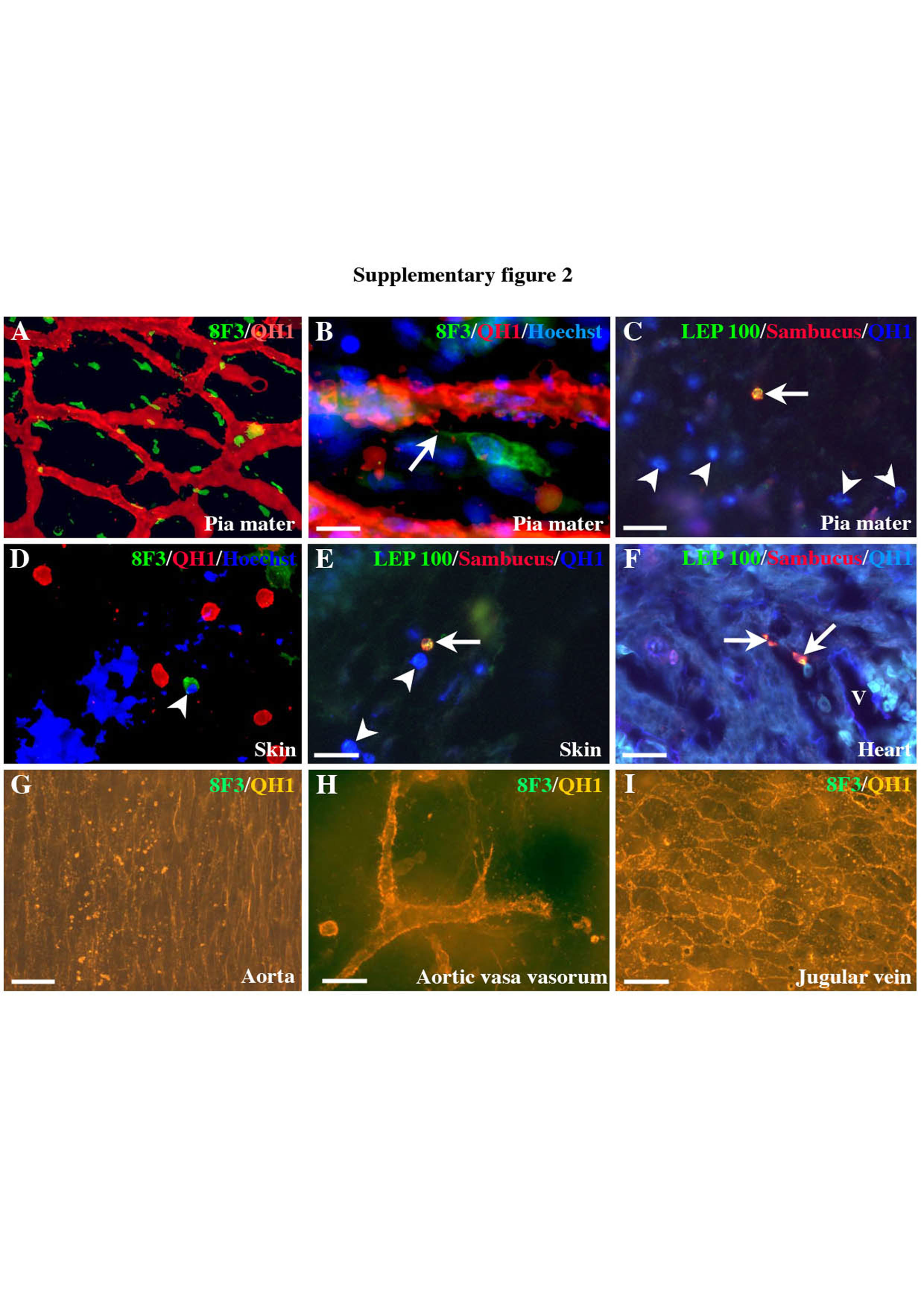

Supplement: Figure S2 — Distribution of chick yolk sac-born macrophages in E15 quail chimeras. A) In toto double immunostaining showing 8F3+ cells (green) invading the QH1+ vascular plexus of the pia mater. Bar: 20 mm. B) High magnification of a 8F3+ cell showing a thin filopodial extension (arrow) towards the vascular plexus. Bar: 7 mm. C) Triple staining on a section through the pia mater identifying a chick QH1−/Sambucus+/LEP100+ macrophage (arrow) among QH1+ HC (arrowheads). Bar: 20 mm. D) In toto double immunostaining in the skin showing a chick cell (arrowhead) present among QH1+ cells. Bar: 20 mm. E) Skin section with a triple staining identifying a chick QH1−/Sambucus+/LEP100+ macrophage (arrow) among QH1+ HC (arrowheads). Bar: 20 mm. F) Transverse section through the heart with two chick QH1−/Sambucus+/LEP100+ macrophages (arrows) close to a QH1+ coronary vessel (V). Bar: 20 mm. G–I) In toto QH1/8F3 double staining in large vessels does not identify 8F3+ chick cells in the quail aortic endothelium (G), the quail aortic vasa vasorum (H) and the quail jugular vein endothelium (I). Note the presence of QH1+ HC at the aortic luminal surface (G, orange dots). Bar: 30 mm in G and I, 10 mm in H. (TIF) [file pone.0025889.s002.tif]

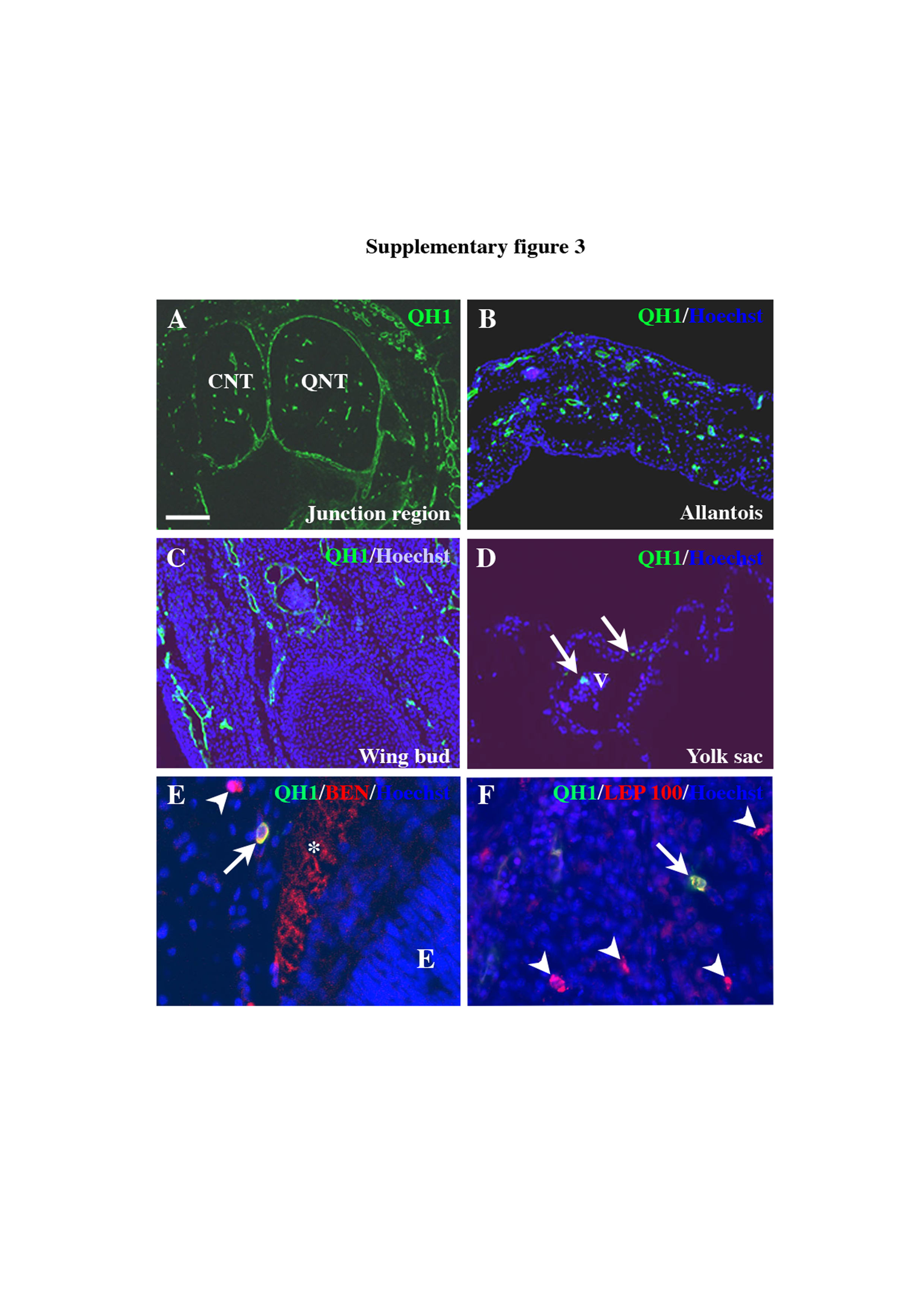

Supplement: Figure S3 — Half embryo chimeras. A) This cross section illustrates the junction region between quail and chick territories where quail (QNT) and chick (CNT) neural tubes overlap. In the quail region QH1+ vessels are present. In the chick territory, a part of the neural tube is vascularized by quail EC that have migrated interstitially. The QH1+ dots on the left are quail HC. B) Section of the quail allantois vascularized by QH1+ vessels. C) QH1+ vessels present in the quail wing bud. D) Section of the chick yolk sac with QH1− vessels in which QH1+ HC (arrows) are observed. E) Presence of a quail QH1+/BEN+ HC (arrow) in the rhombencephalic mesenchyme together with a chick QH1−/BEN+ HC (arrowhead), probably a macrophage. Note that BEN stains the neuronal plexus (*) in the epithelium (E). F) LEP100+ macrophages detection in the diencephalic mesenchyme. One double stained QH1+/ LEP 100+ quail macrophage (arrow) is seen among chick ones (arrowheads). Bar: 40 mm in A–F. (TIF) [file pone.0025889.s003.tif]
